# Measles Cases during Ebola Outbreak, West Africa, 2013–2106

**DOI:** 10.3201/eid2306.161682

**Published:** 2017-06

**Authors:** Francesca Colavita, Mirella Biava, Concetta Castilletti, Serena Quartu, Francesco Vairo, Claudia Caglioti, Chiara Agrati, Eleonora Lalle, Licia Bordi, Simone Lanini, Michela Delli Guanti, Rossella Miccio, Giuseppe Ippolito, Maria R. Capobianchi, Antonino Di Caro, the Lazzaro Spallanzani Institute for Research and Health Care Ebola Virus Disease Sierra Leone Study Group

**Affiliations:** Lazzaro Spallanzani Institute for Research and Health Care, Rome, Italy (F. Colavita, M. Biava, C. Castilletti, S. Quartu, F. Vairo, C. Caglioti, C. Agrati, E. Lalle, L. Bordi, S. Lanini, G. Ippolito, M.R. Capobianchi, A. Di Caro);; Emergency Nongovernmental Organization, Milan, Italy (M. Delli Guanti, R. Miccio)

**Keywords:** measles, measles virus, viruses, Ebola virus disease, outbreak, Ebola virus, IgM, Sierra Leone, West Africa

## Abstract

The recent Ebola outbreak in West Africa caused breakdowns in public health systems, which might have caused outbreaks of vaccine-preventable diseases. We tested 80 patients admitted to an Ebola treatment center in Freetown, Sierra Leone, for measles. These patients were negative for Ebola virus. Measles virus IgM was detected in 13 (16%) of the patients.

The Ebola virus disease (EVD) outbreak in West Africa during 2013–2016 was one of the worst public health disasters in recent history; it caused >28,646 cases and 11,323 deaths (*1*). Consequences of EVD include social instability, poor food reserves, breakdown of healthcare systems, and reduced vaccination coverage (*2*,*3*). Breakdown of healthcare systems and reduced vaccination coverage might have been the worst consequences because nearly all health resources were shifted to the EVD response. Disruptions of local health systems could lead to underreporting of other infectious diseases cases and a second crisis that could kill as many as persons as the original outbreak, if not more.

The 3 countries most affected by this outbreak (Sierra Leone, Guinea, and Liberia) have been a major part of the World Health Organization Expanded Programme on Immunization through vaccination campaigns for reducing childhood deaths from vaccine-preventable diseases, such as measles. Although there are no cures for EVD or measles, a potent measles vaccine is available, which can prevent spread of this disease. Use of this vaccine is crucial because measles is far more contagious (1 case-patient with measles can transmit infection to 12–18 persons) than EVD and might be the primary cause of major epidemics (*3*,*4*). These 3 countries reported nearly 93,685 cases of measles during 1994–2003 (although Sierra Leone did not report cases for 4 years), and during November 1, 2009–July 13, 2010, a total of 1,094 confirmed measles cases were reported in Sierra Leone. Plans for measles vaccination campaigns were implemented before the EVD outbreak because of an increase in susceptibility to measles in these 3 countries (*3*,*5*).

Historically, measles outbreaks have followed humanitarian crises, such as war (*6*), natural disasters (*7*), and political crises (*8*). Recent studies have shown that measles is one of the causative agents of secondary outbreaks during the EVD epidemics in West Africa (*9*), probably due to the disruption in vaccination campaigns, nonfunctional healthcare systems (including detection and reporting of measles cases), lack of specific treatment, and a sense of fear of contracting EVD with reluctance to approach health assistance (*10*).

Probable underreporting of and lack of data for measles cases during EVD outbreaks prompted us to investigate measles in Sierra Leone during the recent EVD outbreak. Although during the preparedness phase of the European Mobile Laboratory Project (http://www.emlab.eu), measles was to be included among diseases tested for differential diagnosis of EVD, we could not implement this approach in the field during the outbreak.

We performed a retrospective serologic study to partially investigate the role of measles in the EVD outbreak by testing serum samples negative for Ebola virus by reverse transcription PCR for measles virus IgM from persons suspected of having EVD. Samples were obtained at the Emergency Nongovernmental Organization Ebola Treatment Center (Goderich, Freetown, Sierra Leone). This study was approved by Ethical Committee of Sierra Leone.

We analyzed 80 patients, of whom 27 were >8 years of age and <25 years of age (median age 30 years) during December 2014–June 2015, who had fever (temperature >37.5°C) and diarrhea or vomiting. Only 1 patient had a history of rash. Measles virus IgM was detected by using an indirect immunofluorescence assay for 13 patients (16%), most (69%) of whom were in this age group (p<0.001). Although we could not determine whether measles IgM in these patients was caused by vaccination failures during the outbreak or failures of the Expanded Programme on Immunization, this age distribution might be indicative of noneffective immunization campaigns, especially a deficiency in the healthcare system in detecting and reporting measles cases.

The recent EVD outbreak caused breakdowns of healthcare systems in the affected countries, leading to possible secondary outbreaks (*2*,*3*). A higher risk for vaccine-preventable diseases, in particular measles, is often an early result in interruption in delivery of public health services. Recent studies have shown that the increase in measles cases during the EVD outbreak in 2013–2016 was caused by disruption of vaccination programs and underreporting of measles cases, which is probably related to effects of EVD on healthcare systems (*9*).

Our results for samples obtained from Ebola-negative patients showed a high number of measles infections during the outbreak in different age groups. Although few (n = 80) patients have been tested, our results provide useful insights into measles cases during other outbreaks in different age groups, adding new evidence from a study that focused on children (*9*).

Our findings indicate the need for correct and rapid differential diagnoses during such outbreaks to avoid spread of other infectious diseases. Furthermore, local public health systems should be strengthened in those countries that are now recovering from the EVD outbreak to reduce risks for other infectious diseases outbreaks.
